# Editorial: Mouse Models of B Cell Malignancies

**DOI:** 10.3389/fimmu.2021.789901

**Published:** 2021-10-28

**Authors:** Gema Perez-Chacon, Christelle Vincent-Fabert, Juan M. Zapata

**Affiliations:** ^1^ Centro Nacional de Investigaciones Oncológicas (CNIO), Madrid, Spain; ^2^ UMR CNRS 7276/INSERM U1262 CRIBL, University of Limoges, Limoges, France; ^3^ Hematology Laboratory of Dupuytren, Hospital University Center (CHU) of Limoges, Limoges, France; ^4^ Instituto de Investigaciones Biomédicas “Alberto Sols”, CSIC-UAM, Madrid, Spain; ^5^ Instituto de Investigación Sanitaria La Paz (IdIPAZ), Madrid, Spain

**Keywords:** genetically engineered mouse models, GEMM, mouse lymphoma, mouse leukemia, B cell neoplasms, immunosurveillance

B cell malignancies represent a vast group of different entities arising from the multiple differentiation stages of B cells. In humans, there is a large variety of B cell malignancies, most of which have counterparts in mice. Prognosis and treatment for these malignancies would be largely dependent on their type, stage and grade.

Malignant B cells, like their normal counterparts, can disseminate around the body and have the remarkable ability to generate clonal diversity by mutation, often resulting in the development of treatment resistances. The microenvironment is crucial to drive tumor evolution, and we cannot yet adequately recapitulate *ex vivo* the complexity of the crosstalk between malignant B cells, normal lymphoid cells and stroma. Mouse models of B cell malignancies offer the possibility to study these complex relations *in vivo*. They also provide insights into the cellular and molecular mechanisms driving tumor evolution and may provide a preclinical platform for testing new therapies against leukemia and lymphoma.

Genetically engineered mouse models (GEMMs) mimicking the alterations in the expression and/or bearing cancer-driving mutations of candidate genes (oncogenes and tumor suppressors) implicated in B cell tumorigenesis are essential tools to assess the role of these genes in cancer. In addition, the unexpected development of B cell malignancies by genetically modified mice helped to uncover neoplastic functions of a variety of genes, unveiling new targets for therapy. Furthermore, the next generation sequencing has opened new possibilities for forward and reverse genetic screenings to identify gene mutations involved in tumor development, progression, evasion and relapse, both in human and mice, shaping the field for an exciting future.

This Research Topic is a collection of 13 articles that provides an overview on the recent developments in the field, including comprehensive reviews on the existing mouse models of B cell leukemia and lymphoma and on the new techniques available to characterize these models. In addition, original research articles describing new mouse models of monoclonal gammopathy of undetermined significance (MGUS), Waldenström macroglobulinemia (WM) and chronic lymphocytic leukemia (CLL) provide new insights into the role of a variety of oncogenes on the development of these B cell neoplasms.

The new methodologies involving forward and reverse genetics and their applications in mouse research to identify new pro-oncogenic gene mutations as drivers of B cell malignancies is reviewed by Uren and Dawes. This timely and comprehensive review focuses on the experimental high throughput genetic approaches as tools to identify and validate large numbers of candidate genes. The authors overview how forward genetic screening in mice using insertional mutagenesis, chemical mutagenesis and exome sequencing can help to identify cooperating mutations at rates not possible using human tumor genomes. Uren and Dawes also address the usefulness of reverse genetic models and screenings that use CRISPR-Cas9, ORFs and shRNAs to provide high throughput *in vivo* proof of oncogenic function.

## New Mouse Models of Plasma Cell Neoplasms

Plasma cell neoplasms are tumor entities composed of post-germinal center (GC), terminally differentiated Ig producing B cells. There are distinct subclasses, including MGUS), WM, plasmacytoma and multiple myeloma (MM). Among those, MM is the most aggressive and the second most frequent leukemia (1), still causing the death of many patients due to the development of treatment resistances. Pisano et al. provide an insightful review of the available mouse models of plasma cell neoplasms, including transplantation-based and transgenic mouse models. The authors discuss the strengths and weaknesses of these mouse models for the study of MM and other plasma cell neoplasms, highlighting past achievement, current developments and future directions in the field aimed to develop new therapies that improve the outcome of patients with MM.

Schmidt et al. and Ouk et al. present original research providing conclusive evidence on the role of constitutive MyD88 activation on the development of plasma cell neoplasm. A mutation in the *MyD88* gene introducing a leucine in position 265 instead of a proline causing constitutive MyD88 dimerization and NFKB and JAK activation is found in a variety of human B cell neoplasms (2), including in most patients with WM (3). Schmidt et al. developed three genetically engineered conditional mouse models harboring floxed-*Myd88^L252P^* (the mouse homolog of the human L265P mutation), one with *Cre* under the control of CD19 (CD19-Cre mice), where Myd88^L252P^ expression is enforced in all B cells, a second mouse strain with *Cre* under the control of Cγ1 promoter (Cγ1-Cre mice), thus limiting *Myd88^L252P^* expression to GC cell, and a third mouse line with restricted *MyD88^L252P^* expression to a few random B cells (CD19-CreERT2 mice). All these models developed distinct manifestations of IgM plasma cell hyperplasia, with the *Myd88^L252P^;CD19-Cre^ERT2^* mice developing monoclonal IgM paraproteins and IgM-expressing plasma cell expansions consistent with MGUS, the premalignant condition preceding WM. In addition, Ouk et al. independently generated a transgenic mice constitutively expressing Myd88^L252P^ in CD19 B cells (*Myd88^L252P^-*IRES*-Yfp*;*CD19-Cre*). They show that these mice accumulate plasma cells in bone marrow and develop serum hyper-gammaglobulinemia, similar to the *Myd88^L252P^*;*CD19-Cre* model described by Schmidt et al. Interestingly, although Schmidt et al. did not observe a path to plasma cell transformation in their model, Ouk et al. performed a longitudinal analysis that show that most of their mice develop with aging a monoclonal IgM peak and spleen lymphoplasmacytic-like B cell lymphoma with a transcriptomic signature consistent with WM. These two excellent works highlight the key role of the *Myd88^L252P^* mutation and constitutive Myd88 activation in plasma cell transformation. Interestingly, the results with the two *Myd88^L252P^;CD19-Cre mice* also raise questions on whether a different microbial and mouse housing environments, and even mouse genetic backgrounds, might influence the transformation outcome.

TNF-Receptor Associated Factor (TRAF)-3 controls Toll-like Receptors (TLR) signaling by recruiting and regulating MyD88 function (4). Remarkably, transgenic mice with enforced expression of TRAF3 in B lymphocytes also cause plasma cell expansions, hypergammaglobulinemia and exacerbated TLR responses (5) In addition, a role of TRAF3 in B cell lymphomagenesis is highlighted by the fact that mouse models with B cell-restricted upregulated TRAF3 expression (6) or TRAF3 deficiency (7) develop post-GC and mostly pre-GC B cell lymphomas, respectively. Looking for the mechanisms involved in TRAF3-deficiency-mediated B cell transformation, Liu et al. have uncovered a new role for TRAF3 in regulating B cell viability. The results provided by Liu et al. indicate that, in response to survival and/or growth factors deprivation, TRAF3 is mobilized to mitochondria, where, through its interaction with MFF, triggers mitochondria-dependent apoptosis. This new role of TRAF3 in controlling mitochondria homeostasis might have key implications in the role of TRAF3 in B cell transformation, providing new insights into why TRAF3-deficient mice (7) and TRAF3xBCL2 tg mice (6) (the later with protected mitochondria by means of BCL2 overexpression) develop distinct types of B cell neoplasia.

## Mouse Models of c-Myc-Driven Lymphomas

c-MYC is a major transcriptional regulator controlling proliferation, cell growth, and apoptosis. Dysregulation of *c-MYC* is a trademark of a variety of B-cell lymphomas, where translocations of this gene lead to overexpression of intact c-MYC protein (8). *c-MYC* translocation is a primary transformation event in Burkitt´s lymphoma but its occurrence as a secondary event in diffuse large B-cell lymphoma, plasmablastic lymphoma, mantle cell lymphoma, or double-hit lymphoma fuels the aggressiveness of these lymphomas. Ferrad et al. overview the various *c-Myc*-driven mouse models of lymphoma focusing on those mouse models of c-myc overexpression regulated by the two main enhancers in the Igh locus, namely, Eµ and 3´RR enhancer. The authors comment on the strengths and limitations of these mouse models with deregulated *c-Myc* expression for the study of lymphoma etiology.

In addition, Malaney et al. also overview the various transgenic mouse models of B cell lymphoma based on c-MYC upregulation, with particular emphasis on the heterogeneous nuclear ribonucleoprotein K (hnRNP K) as a driver of B cell lymphoma through its role on c-Myc regulation. hnRNP K is a ssDNA and RNA binding protein that regulates a plethora of processes controlling transcription and translation (9) and its over- and under-expression is causative of cancer (10). hnRNP K has been shown to be upregulated in DLBCL and Burkitt´s lymphoma patients and the oncogenic role of *hnRNP K* was previously confirmed by the authors by means of a B cell specific Eµ-*hnRNP K* transgenic mice that develop B cell lymphomas with high latency and high incidence (11). hnRNP K’s oncogenic potential stems from its ability to regulate c-MYC expression at post-transcriptional and translational level, without requiring *c-MYC* translocations. In this review, Melaney et al. discuss the usefulness of the Eµ-*hnRNP K* mouse models in the study of B cell malignancies and as a preclinical platform for the assessment of novel therapeutics.

## Mouse Models of FL and GCB-DLBCL

Mossadegh-Keller et al. and Meyer et al. provide two comprehensive reviews on the available mouse models of GC-derived B cell lymphomas, which are the most frequent lymphoma types in humans. Mossadegh-Keller et al. pay particular attention to follicular lymphoma (FL) and the GC B cell-like subtype of DLBCL, providing a review of the various mouse models of FL and GCB-DLBCL and their respective key genetic and epigenetic characteristics. The authors overview the role of 1) apoptosis dysregulation, as indicated by the fundamental role of *Bcl2* dysregulation in FL and GCB-DLBCL lymphomagenesis, 2) epigenetic dysregulation (i.e. *Ezh2, Crebbp, Kmt2D*), 3) the tumor microenvironment as a driver of tumor progression and evasion from the immune system, and 4) the metabolism adaptations fueling tumor progression. Meyer et al. provide a thorough overview on the mouse models of Burkitt´s lymphoma, FL and DLBCL based on the key oncogenic events found in the human counterparts of these tumor subtypes, highlighting the pros and cons of each of these mouse models in the context of human disease and their potential therapeutic utility. Meyer et al. also review the different techniques currently available for creating a GEMM and comment on the development of patient-derived xenografts (PDXs) and their usefulness in lymphoma research.

## Mouse Models of Chronic Lymphocytic Leukemia

CLL remains as the most common leukemia in Western countries. CLL is an incurable disease with a variable clinical course consisting of at least two distinct subtypes based on the mutational status of the immunoglobulin heavy chain variable IGHV genes, which could be mutated (M) (good prognosis) or unmutated (UM) (bad prognosis) (12). The establishment of CLL PDXs have been challenging, in part because CLL cells, unlike many other B cell neoplasms, are low-proliferating cells, mostly quiescent and with dysregulated apoptosis. Only a small percentage are proliferating cells, which makes difficult their expansion in immunodeficient mice. Patten et al. provide a thorough analysis of the critical parameters supporting engraftment and growth of patient´s CLL cells in NGS immunodeficient mice. The authors find that *in vitro* pre-activation of CLL-derived T cells is key to support reliable implantation of CLL cells in a fully autologous system. The authors also show that patient´s CLL and T cells implantation follow distinct dynamics that could be differentiated in 4 temporal phases. The usefulness of this PDX approach is illustrated by assessing the effects of a bispecific antibody reactive with B and T cells.

Due to the difficulties in stablishing reliable CLL PDXs as described above, an intense effort has been invested in developing CLL mouse models (13). The most profusely studied CLL mouse model, the *Eµ-TCL-1* transgenic (14), as well as other reported mouse CLL models, only develop UM CLL, thus leaving unrepresented the M-CLL subtype. Another mouse model of CLL is the *Traf2*DN/*BCL2*-double-tg mice, that develop CLL/Small Lymphocytic Lymphoma (SLL) with high penetrance with aging. This mouse model has unbridled BAFF signaling and constitutive NFKB2 activation, causing the expansion of marginal zone (MZ) B cells (15) and, together with BCL2 overexpression, which is a CLL trademark, predispose MZ B cells to transformation into CLL/SLL. Perez-Chacon and Zapata provide original research showing that the CLL/SLL arising in the *Traf2*DN/*BCL2*-tg double-tg mice consists of both expanded M- and UM-CLL/SLL clones. Expanded clones show a biased IGHV gene usage, stereotypy and express HCDR3 that are similar to those recognizing autoantigens and pathogen antigens, thus closely resembling human CLL.

## Pathogens and Immune Evasion in Lymphomagenesis

The role of pathogens in promoting B cell transformation is reviewed by Huang and Yasuda, who focus on the role of Epstein-Barr virus (EBV) infection in lymphomagenesis. EBV is endemic in humans, with approximately 95% of the world’s population sustaining an asymptomatic life-long infection. EBV may cause a variety of immune diseases in immunosuppressed individuals, including tumorigenesis when infected cells evade immunosurveillance (16). Huang and Yasuda summarize the role of EBV proteins and RNAs in promoting lymphomagenesis, the types of EBV-associated lymphomas and the available mouse models to study EBV-driven lymphoma.

The key role of immunosurveillance in preventing tumorigenesis is addressed by Lemasson et al., who evaluates how GEMMs could help in assessing the specific role of the distinct immune checkpoints in immunosurveillance and how B cell lymphomas scape from their control. The authors review the mouse models of B cell lymphoma that have been used to study the involvement of the PD-1/PD-L1 axis, CTLA-4, MHC-II and NKG2 in lymphoma immune evasion. The authors also overview the relevance for the human disease of these mouse models and the usefulness of these models to pharmacologically target these checkpoint molecules to improve current treatments.

Overall, the original articles and reviews contained in this Research Topic provide a broad and updated view on the current GEMMs of B cell malignancies, highlighting the essential role of genetically modified mice in understanding all aspects of B cell lymphoma and leukemia, including development, progression, immune evasion and evolution to refractory disease.

## Author Contributions

All authors listed have made substantial, direct and intellectual contribution to the article and approved it for publication.

## Funding

GP-C and JMZ were supported by a grant from the Agencia Estatal de Investigacion (PID2019-110405RB-I00/AEI/10.13039/ 501100011033). CV-F was supported by the France Lymphome Espoir association of patients and the Ligue contre le cancer.

## Conflict of Interest

The authors declare that the research was conducted in the absence of any commercial or financial relationships that could be construed as a potential conflict of interest.

## Publisher’s Note

All claims expressed in this article are solely those of the authors and do not necessarily represent those of their affiliated organizations, or those of the publisher, the editors and the reviewers. Any product that may be evaluated in this article, or claim that may be made by its manufacturer, is not guaranteed or endorsed by the publisher.
